# Efficacy and Tolerability of Malartin and Sulphadoxine-Pyrimethamine Combination against Uncomplicated Falciparum Malaria in Dibanda, Southwest Cameroon

**DOI:** 10.1155/2012/372518

**Published:** 2012-02-29

**Authors:** Helen Kuokuo Kimbi, Mesame Ntoko, Nelson N. Ntonifor, Emmaculate Lum, Anna L. Njunda, Peter Nde Fon

**Affiliations:** ^1^Department of Plant and Animal Sciences, Faculty of Science, University of Buea, P.O. Box 63, Buea, SWR, Cameroon; ^2^Department of Biochemistry and Microbiology, Faculty of Science, University of Buea, P.O. Box 63, Buea, SWR, Cameroon; ^3^Department of Medical Laboratory Science, Faculty of Health Sciences, University of Buea, P.O. Box 63, Buea, SWR, Cameroon; ^4^Department of Public Health, Faculty of Health Sciences, University of Buea, P.O. Box 63, Buea, SWR, Cameroon

## Abstract

Artemisinin derivatives are now the most potent and rapidly acting antimalarials. The aim of this study was to assess the *in vivo* efficacy and tolerability of a combination of Malartin (an artesunate) and sulphadoxine-pyrimethamine (SP) in the treatment of uncomplicated falciparum malaria in Dibanda, Cameroon. A total of 197 subjects were recruited into the study and administered Malartin for 3 days and SP as a single dose on day 0. Only 174 of the subjects were successfully followed up on days 3, 7, and 14. The overall success rate of the drug combination was 92.53%. Parasite density decreased during the follow-up period in different age groups, sexes, and social classes. The prevalence of anaemia decreased from 22.99% at enrolment to 9.77% on day 14, and the difference was significant (*P* < 0.05) on all days of followup. The drug combination did not give rise to any serious side effects.

## 1. Introduction

Malaria continues to be a debilitating disease with public health consequences on an ailing population that is already poor [1]. Many malaria control strategies exist, but none is appropriate and affordable in all contexts, especially in endemic areas. The World Health Organization (WHO) [2] recommends three essential elements of malaria control, namely, the application of vector control, early detection, or forecasting of epidemics, and early diagnosis and prompt treatment of the disease.

*Plasmodium falciparum* is the main infecting species and its infections can be very fatal unless promptly treated. It is responsible for the majority of deaths due to malaria and the situation has been worsened by the development of resistance by this parasite to most antimalarial drugs [3]. Resistance to antimalarial drugs arises as a result of spontaneously occurring mutations that affect the structure and activity at the molecular level of the drug target in the malaria parasite or affect the access of the drug to that target [4]. Drug resistance has been implicated in the spread of malaria to new areas and reemergence of malaria in areas where the disease had been eradicated resulting in increased morbidity and mortality [5]. Hence it is important to ensure a rational use of the few remaining effective drugs in order to maximize their useful therapeutic life without sacrificing the safe, effective, accessible, and affordable treatment of those at risk. As such there has been a reexamination of the potential of combinations of existing products and the development of new drug combinations. The WHO [4] now recommends the use of artemisinin-based combination therapies (ACTs) for the first-line treatment of *P. falciparum *in endemic areas, since resistance may be delayed or prevented by using combinations of drugs with independent modes of action and different biochemical targets in the parasite. Artemisinin or one of its derivatives is usually used with one or more other longer acting partner drug(s) in a treatment regimen that can protect against recrudescent infections. The rapid onset of action of artemisinins reduces the biomass of the parasite within a short period of time and the remaining parasites are eliminated by the longer-acting partner drug [4]. Cameroonian health authorities adopted this WHO policy for the treatment of uncomplicated malaria in 2004.

The artesunate, Malartin, (Kakwa Biofarm) was recently introduced into the Cameroon market and its efficacy alone and in combination with amodiaquine has been reported elsewhere [6]. Since its introduction into the market malartin's efficacy in combination with sulphadoxine-pyrimethamine (SP) has not yet been done in the Cameroonian population against uncomplicated falciparum malaria. Although the malartin/amodiaquine combination is effective in eliminating parasites, many patients, however, complain about the side effects of the amodiaquine component of the drug combination. There is therefore a need to evaluate other combinations with malartin in the Cameronian population in order to know the best combination that is most efficacious and safe. This study was therefore undertaken to assess the efficacy and tolerability of a combination of Malartin and SP against uncomplicated *P. falciparum* in Dibanda, Southwest Cameroon.

## 2. Materials and Methods

### 2.1. Study Site

This work was carried out in Dibanda village, southwest Cameroon. The centre for recruiting and treating patients was the Cameroon Christian Medical Foundation (CAMEF). Dibanda has a population of about 3500 people and is located on the eastern slope of Mount Cameroon, where the dense equatorial forest has been cleared. Weather records from the Cameroon Development Corporation indicate a mean relative humidity of 83.1%, temperature range of 18°C to 35°C and an annual rainfall of 4000 mm [7]. There is intense and perennial *P. falciparum* transmission in the area.

### 2.2. Study Patients

The study was carried out between December 2007 and August, 2008. Patients who presented with signs and symptoms of malaria at CAMEF were referred to members of the research team after consultation. The reason for the study was explained to them before asking for their written informed consent. Participants were eligible for inclusion into the study if they were ≥1 year, diagnosed with monoinfections of uncomplicated *P. falciparum* malaria, parasitaemia ≥1000 asexual parasites/*μ*L blood, absence of concomitant illness, and written informed consent by the patient or legal guardian in the case of minors. Those excluded from the study were all participants who took antimalarial drugs in the previous 48 hours, pregnant and lactating women, those with severe malaria or any danger signs as defined by the WHO or mixed infections of plasmodia, and those who refused to participate in the study. Voluntary withdrawal, development of severe manifestations following therapy and overt toxicity (hypersensitivity) to the drugs were considered reasons enough for withdrawing patients from the study. They were then treated with oral quinine (25 mg body weight per day for 5 days). An ethical clearance for this work was obtained from the Ethical Review Committee of the Delegation of Public Health, South West Region, Cameroon. Overall, 576 patients were screened, amongst whom 197 were eligible for inclusion in the study.

### 2.3. Collection and Processing of Blood Samples

Before blood collection, demographic data such as age, gender, social class (poor class—those who lived in plank houses, used pit toilets, and were either unemployed or subsistence farmers with no steady income; middle class—those who lived in cement block houses, used flush toilets, and had basic amenities such as television, refrigerator and radio, and earned a steady monthly salary; rich class—those who had all what the middle class individuals had or more as well as a car), weight, prior use of antimalarial drugs, and clinical manifestations were recorded. Blood samples were obtained through a finger prick for the preparation of thick and thin blood films and determination of packed cell volume (PCV). Blood films were Giemsa-stained according to the method of Cheesbrough [8] and observed for malaria parasites using the oil immersion (x100) objective of the microscope. The thick films were used to determine the levels of parasite density by counting the asexual forms of parasites found per 200 white blood cells (WBC) assuming a WBC count of 8,000 WBC/*μ*L blood [8]. This was used to calculate the geometric mean parasite density (GMPD). Slides were considered negative if no parasites were seen in 100 high-power fields of the microscope. The thin films were used to check that the malaria parasites detected were monoinfections of *P. falciparum* [8]. In order to double-check the results, slides were examined by two experienced microscopists independently.

### 2.4. Determination of Packed Cell Volume (PCV)

Blood-filled capillary tubes were spun at 12,000 *g* for 5 minutes and PCV values were read using a PCV reader. Patients were considered to be anaemic if PCV values were <31%.

### 2.5. Treatment and Followup of Participants

Each patient was treated with 4 mg/Kg body weight/day of malartin on days 0, 1, and 2 and 25 mg/kg body weight of sulphadoxine and 1.25 mg/Kg body weight of pyrimethamine in a single dose [4]. The first dose of the drugs was administered under direct supervision and patients were observed for 30 minutes for vomiting and allergic reactions to the drugs. If a patient vomited the treatment was repeated. If vomiting persisted, the patient was withdrawn from the study and placed on quinine therapy. The day of sampling was termed day 0. Patients were told to come back to the clinic on days 3, 7, and 14 for followup. The side effects experienced by the patients were recorded, on days of followup, and they were encouraged to return at any time they felt ill or in case of clinical deterioration. All the individuals who failed to come back on subsequent appointment days were visited at home. In cases where the patients could not be located at all, they were excluded from the study.

The blood of the patients was collected on all days of followup to determine their parasite density and PCV levels. Study participants were considered to have attained a study endpoint whenever there was onset of symptoms requiring alternative drugs and/or hospitalization, receipt of another antimalarial from outside the study team, or voluntary withdrawal. Participants still parasitaemic on day 14 were referred for alternative treatment.

### 2.6. Safety Assessment

Any signs and symptoms that first occurred or became more severe after treatment were considered to be the adverse effects of treatment and were recorded during the course of followup.

### 2.7. Therapeutic Endpoints

Treatment outcome was classified as early treatment failure (ETF), late clinical failure (LCF), late parasitological failure (LPF), or adequate clinical and parasitological response (ACPR), according to the WHO [9] criteria albeit with a day 14 endpoint instead of a day 42.

(i) To be categorized as an ETF, a patient had to show danger signs or severe malaria on days 1, 2, or 3, in the presence of parasitaemia; a day 2 parasitaemia that was greater than the day 0 (irrespective of axillary temperature); a parasitaemia on day 3, with fever (an axillary temperature of at least 37.5°C); or a day 3 parasitaemia that was at least 25% of the day 0.

(ii) To qualify as LCF, the patient had to show danger signs or severe malaria, in the presence of parasitaemia, on any day after day 4 and before day 14, without previously meeting any of the ETF criteria; or fever in the presence of parasitaemia on any day between day 4 and day 14, without previously meeting any of the ETF criteria.

(iii) Any patient found parasitaemic between day 4 and day 14, but with a temperature of <37.5%, without previously meeting any of the criteria of ETF or LCF, was considered as an LPF.

(iv) All other patients who were aparasitaemic on day 14, irrespective of axillary temperature, were considered ACPR.

### 2.8. Statistical Analysis

The data were analyzed using Microsoft Excel 2002 and SPSS version 10. Students' *t-*test or analysis of variance (ANOVA) was used to test the differences between the treatment means. The chi square test or Fisher's exact *χ* ^2^ was used for comparisons of the cure rates. The level of significance was set at *P* < 0.05.

## 3. Results

### 3.1. Baseline Characteristics of Patients

Overall, 576 people aged 1 to 78 years were screened and a total of 197 participants satisfied the enrolment criteria and were included in the study. Twenty-three patients were lost in the course of followup. A total of 174 participants were successfully followed up (74 males and 100 females). The median age was 11.4 (Table 1). Overall, 78.16% (136/174) of the participants were of the poor class while there were 32 and 6 patients in the middle and rich classes, respectively.

### 3.2. Assessment of Drug Efficacy

The adequate clinical and parasitological response (ACPR) rate on day 14 was 92.53% (161/174). The early treatment failure (ETF) rate was 0.60% (01/174), while the late clinical failure (LCF) and late parasitological failure (LPF) rates were 03.45% (06/174) and 03.45% (06/174), respectively. Overall, the clinical treatment failure rate was 07.50% (Table 2).

### 3.3. Variation in Geometric Mean Parasite Density with respect to Sex, Age, and Social Class of Patients

 There was a steady decrease in GMPD in both sexes from day 0 to day 14, but the difference was insignificant except on day 7 (Table 3) when the GMPD in males was significantly higher than that in females (*χ* ^2^ = 4.826, *P* = 0.03). GMPD decreased steadily in all age groups from day 0 to day 14 following treatment (Table 3), but the difference was only significant on day 0 (*F* = 3.667, *P* = 0.014). There was no significant difference in GMPD across the different social classes during followup after treatment with Malartin and SP (Table 3).

### 3.4. The Effect of Malaria Parasitaemia on Anaemia Status during Followup

At enrolment, 22.99% (40/174) of the participants were anaemic and had a higher GMPD when compared with the nonanaemic subjects (Table 4). This GMPD was observed to decrease steadily from day 0 to day 14. There was no significant difference in GMPD between the two groups, except on day 0 when the difference was significant (*χ* ^2^ = 6.528, *P* = 0.002).

### 3.5. Prevalence of Anaemia in the Different Age Groups

77.01% (134/174) of participants were not anaemic at enrolment. Anaemia was generally more prevalent in children (<15 years) than in adults (≥15 years), and the difference was significant (*P* < 0.05) on all days of followup (Table 5). The proportion of anaemic subjects (both children and adults) decreased from 22.9% on day 0 to 9.77% on day 14 and the difference was significant (*χ* ^2^ = 10.41, *P* = 0.034).

### 3.6. Prevalence of Anaemia in the Different Social Classes

 Overall, anaemia was more prevalent in the middle class, and generally decreased gradually in all the different social classes after treatment with Malartin and Fansidar. On day 14, the highest prevalence (10.30%) was recorded in the poor class. There was no significant difference in the prevalence of anaemia across the different social classes during followup after treatment with Malartin and Fansidar (*P* > 0.05).

### 3.7. Variation of Body Temperature during Followup

It was observed that after administering malartin and SP to the subjects, their body temperatures reduced rapidly and fever clearance on day 3 was 97.00%. Variation of body temperature during followup was done by comparing the mean temperature on day 0 with that of the rest of the followup days (data not shown). The mean body temperature on day 0 (37.91 ± 0.93) was significantly higher than that of day 14 (37 ± 0.08) (*F* = 53.10, *P* = 0.001).

### 3.8. Assessment of Drug Safety during Followup

A total of 40 out of 174 subjects experienced side effects, and fatigue (25.00%), fever (22.50%), and dizziness (17.50%) were the most prevalent side effects. These side effects were generally minor and self-limiting, and most of them disappeared by day 7.

## 4. Discussion

This study addresses drug-resistant malaria, which has become one of the most important problems in malaria control in recent years especially as it has been extremely difficult to develop an effective antimalarial vaccine and presently there is no single method of control that can completely eliminate malaria. According to WHO [4], the fight against antimalarial drug resistance and its future can be defined by a number of assumptions. First, there is no control or preventive measure at the moment that can totally eradicate malaria, so antimalarial drugs will be needed for a very long time to come. Secondly, so long as drugs are being used, there is a very high probability for resistance to develop, and unfortunately the development of new drugs seems to be taking longer than the development of resistance. Affordability remains a very important factor when considering any strategy to control drug-resistant malaria, especially in Africa. It is therefore very important to find ways and means to maintain the efficacy of the existing antimalarial drugs for as long as possible, since prompt treatment remains the most effective means of control at the moment. *P. falciparum* has been reported to have a complex genome, and is responsible for over 90% of the malaria cases transmitted in southwest Cameroon [7, 10]. Incomplete parasitological cure may lead to anaemia or the return of clinical illness which can progress to severe disease [11]. An optimal antimalarial drug should therefore succeed in achieving clinical cure, and in clearing parasites and maintaining a parasite-free period for as long as possible [4].

In this study, the ACPR at day 14 was 92.53% following treatment with Malartin and SP. This study therefore demonstrated that a combination of Malartin and SP is good for the treatment of uncomplicated *P. falciparum* infection. The observed day 14 ACPR of 92.53% is similar to results of previous studies in Cameroon, where ACPR with a combination of artesunate and Fansidar on day 14 was 93.00% [12]; 93.80% for Malartin and amodiaquine [6] and 91.7% for artesunate and Fansidar [13].

A clinical treatment failure rate of 07.50% was recorded in this study and this may be attributed to the presence of resistant genes (dihydrofolate reductase, *dhfr*) to SP. SP has been widely used in Cameroon as a monotherapy, and is still available in the saturated pool of antimalarials in the country. A study by Mbacham et al. [14] in Cameroon showed that SP has largely depreciated as a monotherapy. The combination of SP with artesunate may likely reduce the rate of emergence of SP and artesunate individual resistances [15]. Artemisinin derivatives are now the most potent and rapidly acting antimalarial drugs to which the malaria parasite is still largely susceptible [16, 17]. Malartin and SP act essentially as blood schizonticides [4]. Malartin like other artesunates treats malaria caused by all species of plasmodia, including multiple drug-resistant strains of *P. falciparum*, and is known to be rapidly schizonticidal, and by suppressing cytokines and the production of tumour necrosis factor (TNF) helps shorten fever-clearance times [4, 18]. On the other hand, SP is effective in eliminating fever, clearing parasitaemia, and improving anaemia in patients [19]. Despite the development of resistance to SP, it might be extremely difficult to eliminate the SP monotherapy from the market as it is cheap, well known, and produced by many generic drug producers. It is also widely used for intermittent preventive treatment in pregnant women in Cameroon.

Other factors that might have contributed to the observed treatment failure rate could include incorrect dosing, noncompliance with the duration of the dosing regimen (since patients had to take the remaining doses of drugs on their own on subsequent days), drug interaction and poor or erratic absorption. Perhaps such factors might have increased the likelihood of parasites being exposed to suboptimal drug levels [4].

The progressive decrease in GMPD in our study indicated that the drugs were effective in reducing the parasite load; there were still some low levels of parasitaemia on day 14 in a few patients. Perhaps this could be due to reinfection, since Dibanda is a highly endemic area for malaria. According to Enevold et al. [20], in malaria endemic areas, children may recover from malaria after chemotherapy in spite of harbouring genotypically drug-resistant *P. falciparum*. This phenomenon suggests that there is a synergy between drug treatment and acquired immunity. In high-transmission areas especially in Africa, the generally accepted objective of malaria treatment is not so much the clearance of parasitaemia but the resolution of clinical symptoms and acute febrile illness as measured by the adequate clinical response and early and late treatment failures [4].

During followup there was also an amelioration in PCV levels. This agrees with other studies in Cameroon, Sudan, Senegal, and Kenya where PCV levels increased following treatment with ACTs [6, 16, 21]. Anaemia is a very serious condition, and when associated with malaria causes morbidity and mortality in young children and pregnant women in malaria endemic areas. This may imply that *P. falciparum* was the major cause of anaemia in the patients, and it is therefore important to monitor the development of drug resistance in this area in order to maintain the therapeutic lifespan of effective drugs for as long as possible. Repeated episodes of malaria due to reinfection or failure to clear parasites as a result of antimalarial drug resistance may result in life-threatening anaemia [22].

In our study, 78.20% of the subjects belonged to the poor class. Anaemia was found to be more prevalent in the middle class patients, and interestingly, there was no significant difference in GMPD across the different social classes during followup. This could be due to our relatively small sample size of the rich and middle classes. This finding could be investigated further with a much larger sample size of the middle and rich classes. It is also worth noting that a possible selection bias might have occurred in the study especially at enrolment of the patients. This is evident as the study subjects were not very representative of the patient population at large. Maybe, for instance, the presence of the medical team attracted those patients who had already suffered a treatment failure with drugs they got from drug stores where patients do not need any medical prescription to buy drugs and self-medication is common. On the other hand, the presence of the investigators and the availability of free diagnostic and treatment facilities during the study period might have attracted to the clinic patients who would otherwise not have come for treatment and this is typical of malaria endemic areas.

Overall, 22.99% (40/174) of the subjects experienced side effects. This is not surprising as artesunate therapy is not known to give rise to any serious side effects [6, 16, 21]. SP on the other hand, has several adverse effects like nausea, vomiting, anorexia, fatigue, dizziness, jaundice, and diarrhoea, and large doses of SP have also been shown to cause haemolytic anaemia and agranulocytosis [23]. In this study, the most frequently reported side effects were fatigue, fever, and dizziness, and these are symptoms that are common with malaria. However, most of the side effects were self-limiting, as most of them disappeared by day 7.

In future, antimalarial therapy may be expanded by combining chemotherapy with vaccines (or other drugs) specifically designed to inhibit transmission of malaria. These “transmission blocking” vaccines or drugs could reduce the potential for onward transmission of gametocytes carrying resistant genes, even if a relatively large number of parasites survive initial treatment. This could work through use of drugs or vaccines with a high degree of specific antigametocidal activity, drugs that nonspecifically reduce the likelihood of gametocytes developing, or drugs or vaccines that interfere with sexual reproduction and infection of the parasite within the mosquitoes when taken up with a blood meal [4].

## 5. Conclusions

This study has shown that malartin and SP are effective and safe against uncomplicated falciparum malaria. This drug combination is therefore a better alternative for the patients that react to malartin/amodiaquine combination in Cameroon. One of the cornerstones of the current approach to malaria control remains the provision of prompt, effective malaria treatment. Finally, malaria control measures, be they directed at the vector or malaria parasites in the human host through mass drug therapy or susceptible hosts by way of chemoprophylaxis, must be coordinated by integrating available technology with an understanding of the epidemiology of the local malaria situation, which includes a consideration of the behaviour of both humans and the vector so as to be able to make the most appropriate and effective interventions.

## Figures and Tables

**Table 1:** Baseline characteristics of patients attending Cameroon Christian Medical Foundation (CAMEF) during the *in vivo* study, 2007-2008.

Characteristic	Value
Number of study subjects	174
Sex ratio (male/female ratio)	74/100
Age (median)	11.4
Age of males (median)	11.8
Age of females (median)	10.9
Body weight (kg) (mean ± SD)	40.40 ± 21.90 (9–95)*
Initial body temperature (°C) (mean ± SD)	37.91 ± 0.93 (36–40)*
Haematocrit (%) (mean ± SD)	34.27 ± 5.64 (13–50)*
% anaemic (PCV < 31%)	22.99% (40/174)
Geometric mean parasite density	4338 ± 11725 (1000–104000)*

*Indicates the range. SD = standard deviation.

**Table 2:** Clinical outcome of patients attending CAMEF during the *in vivo* study, 2007-2008 after treatment with Malartin and SP.

Parameter	Clinical outcome
ACPR	92.53% (161)
ETF	0.60% (01)
LCF	03.45% (06)
LPF	03.45% (06)
**Clinical treatment failure**	**07.50% (13)**

**Table 3:** Variation in geometric mean parasite density of patients attending CAMEF during the *in vivo* study, 2007-2008 by sex, age group, and social class during followup after treatment with Malartin and SP.

Characteristic	GMPD				
	Category	Day 0	Day 3	Day 7	Day 14
Sex	Male *N* = 74	4599 (1014–04000)	2199 (382–5846)	900 (207–4938)	808 (280–3749)
	Female *N* = 100	4154 (1000–42974)	1904 (196–10667)	641 (143–4525)	626 (167–3574)
Level of significance		*F* = 1.418 *P* = 0.235	*F* = 0.625 *P* = 0.430	*F* = 4.826 *P* = 0.030	*F* = 0.764 *P* = 0.386

Age group (years)	1–5 (*N* = 49)	6517 (1087–48511)	2189 (196–5846)	788 (181–3629)	751 (170–3749)
	6–10 (*N* = 33)	3820 (1067–104000)	1652 (203–7719)	608 (182–1920)	604 (475–776)
	11–15 (*N* = 24)	4054 (1200–18316)	2424 (333–7385)	832 (207–2913)	613 (167–2222)
	>15 (*N* = 68)	3524 (1000–36596)	1975 (218–10667)	730 (143–4938)	692 (150–3176)
Level of significance		*F* = 3.667 *P* = 0.014	*F* = 1.232 *P* = 0.300	*F* = 0.740 *P* = 0.540	*F* = 1.143 *P* = 0.342

Social class	Poor class (*N* = 136)	4495 (1000–48511)	2021 (203–10667)	727 (143–4938)	854 (167–3749)
	Middle class (*N* = 32)	4238 (1014–104000)	2239 (196–15846)	838 (385–2939)	746 (350–3176)
	Rich class (*N* = 06)	2193 (1023–5500)	822 (406–2016)	353 (353–353)	0
Level of significance		*F* = 0.793 *P* = 0.454	*F* = 1.315 *P* = 0.272	*F* = 0.282 *P* = 0.755	*F* = 0.234 *P* = 0.792

**Table 4:** Effect of parasitaemia on anaemia in patients attending CAMEF during the *in vivo* study, 2007-2008 following treatment with Malartin and SP.

Status	GMPD (range)			
	Day 0	Day 3	Day 7	Day 14
Anaemic	5948 (1037–104000)	1974 (196–15846)	690 (181–2667)	598 (340–2222)
Non anaemic	3710 (1000–48511)	2005 (203–10667)	747 (143–4938)	624 (167–3749)
Level of significance	*F* = 6.528 *P* = 0.002	*F* = 0.954 *P* = 0.388	*F* = 0.745 *P* = 0.477	*F* = 1.963 *P* = 0.152

**Table 5:** Prevalence of anaemia in different age groups of patients attending CAMEF during the *in vivo* study, 2007-2008, during followup after treatment with Malartin and SP.

Age group (years)	Number of anaemic cases (%)			
	Day 0	Day 3	Day 7	Day 14
1–5 *N* = 49	22 (44.90)	18 (36.73)	13 (26.53)	10 (20.41)
6–10 *N* = 33	06 (18.18)	05 (15.15)	03 (9.09)	01 (3.03)
11–15 *N* = 24	06 (25.00)	03 (12.50)	02 (8.33)	03 (12.50)
>15 *N* = 68	06 (8.82)	5 (7.35)	04 (5.88)	03 (4.41)
Total *N* = 174	40 (22.99)	31 (17.82)	22 (12.64)	17 (9.77)
Level of significance	*χ* ^2^ = 21.48, *P* < 0.001	*χ* ^2^ = 17.69, *P* = 0.001	*χ* ^2^ = 12.15, *P* = 0.016	*χ* ^2^ = 10.41, *P* = 0.034
